# Supplementary material for: A broadly neutralizing anti-influenza antibody reveals ongoing capacity of haemagglutinin-specific memory B cells to evolve
Source: Nat Commun. 2016 Sep 13;7:12780. doi: 10.1038/ncomms12780 (PMC5027281; doi:10.1038/ncomms12780)
Supplement: Supplementary Information — Supplementary Figures 1-11 and Supplementary Tables 1-3 [file ncomms12780-s1.pdf]

Supplementary Information

Supplementary Figure 1

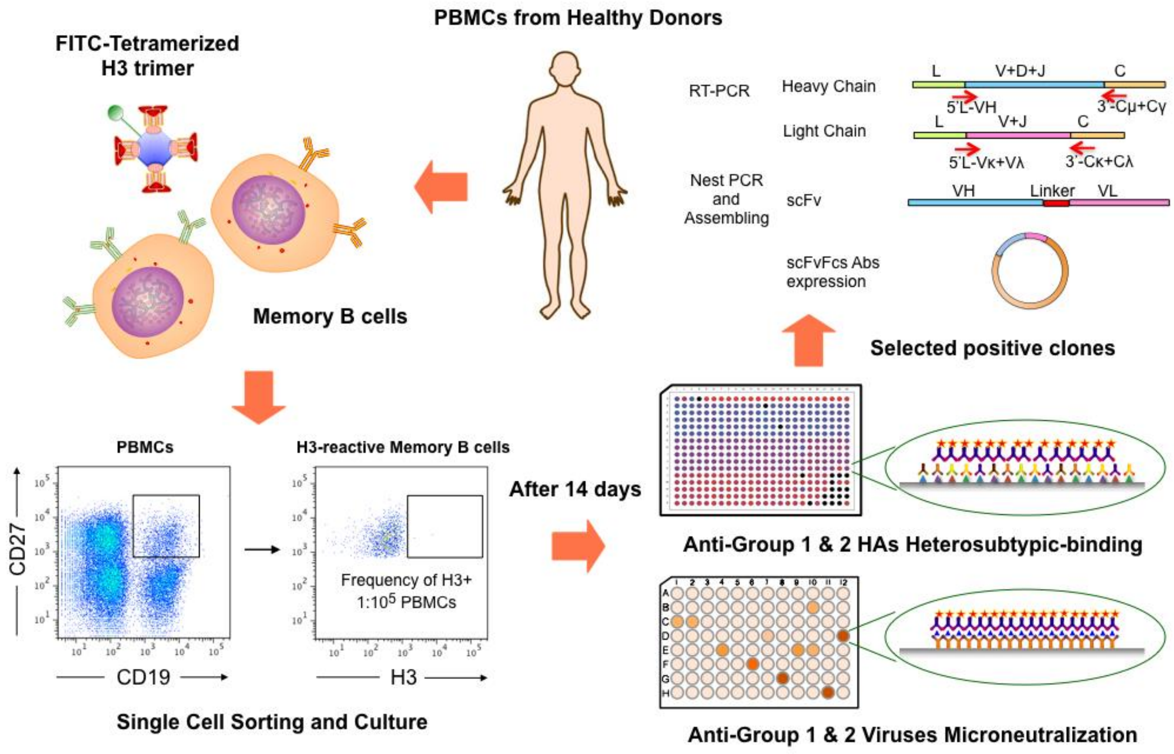

Supplementary Figure 1 Scheme of isolating broadly neutralizing Abs against influenza viruses from human memory B cell repertoire. Representative fluorescence-labeled cell sorting (FACS) data showing frequency of H3-reactive memory B cells from total PBMCs.

Supplementary Figure 2

a

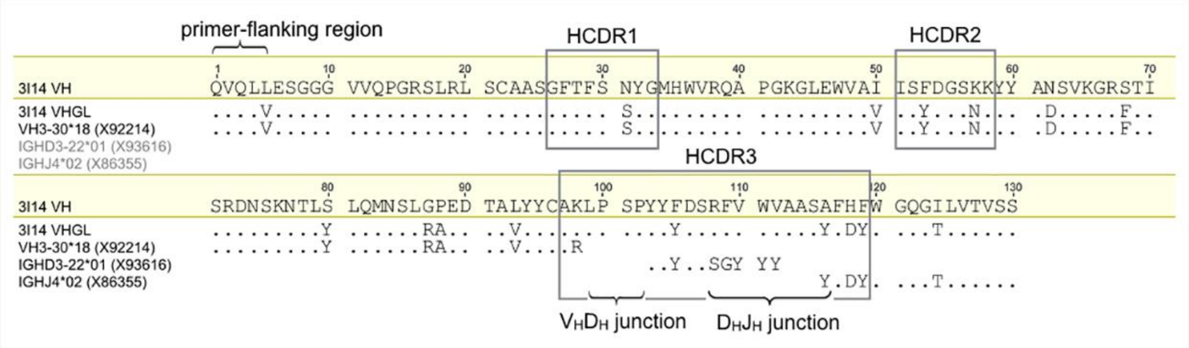

b

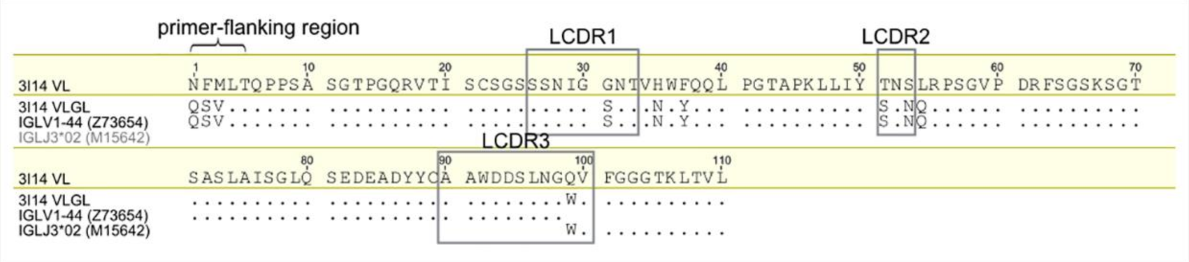

Supplementary Figure 2 Amino acid sequence alignments of 3I14 and related germline heavy chain (**a**) and light chain (**b**) variable region. The corresponding V, D and J sequences determined using the IMGT database are shown for comparison.

Supplementary Figure 3

H1-CA09

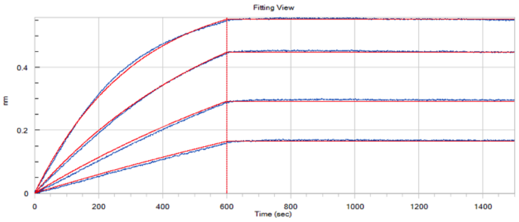

H1-SI06

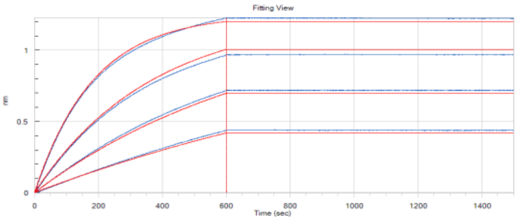

H1-PR8

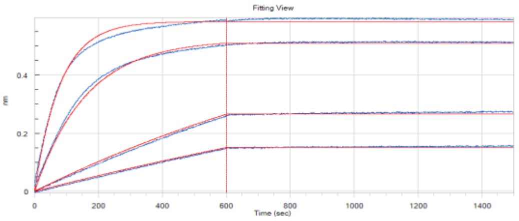

H5-VN04

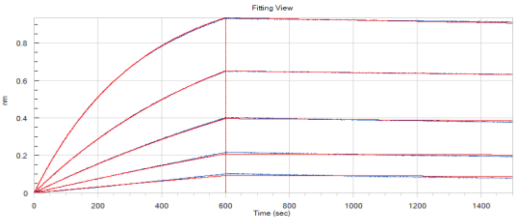

H5-IN05

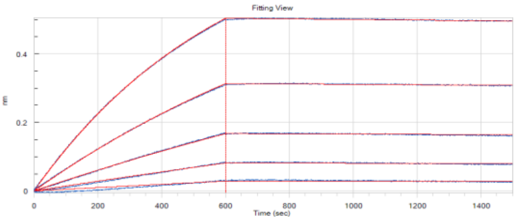

H9-HK99

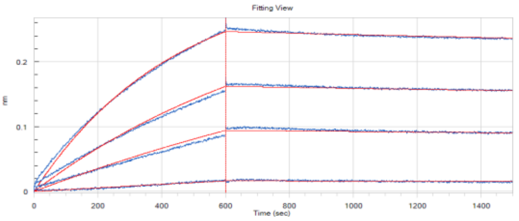

H3-PE09

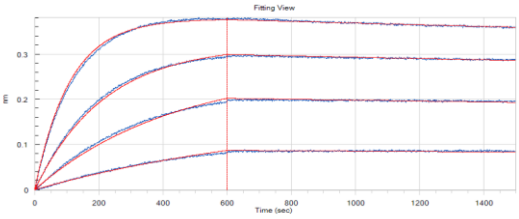

H3-UY07

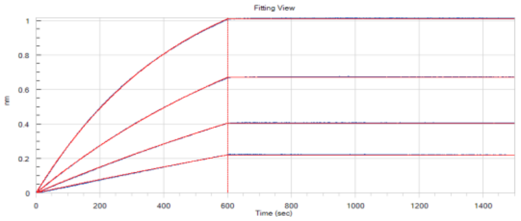

H3-VIC11

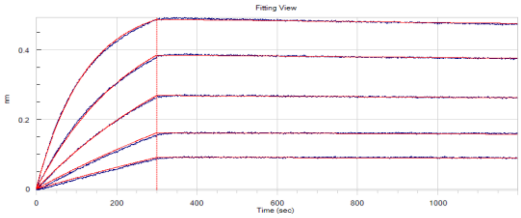

H4-NL05

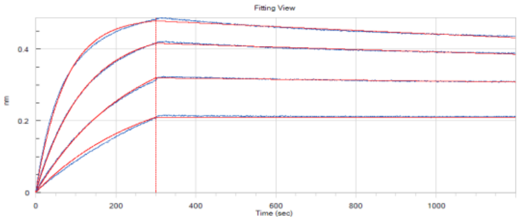

H7-CA444

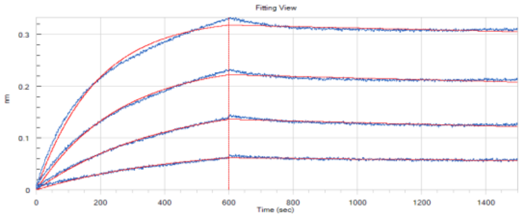

H7-AH13

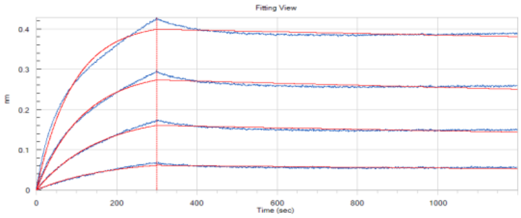

H7-NL219

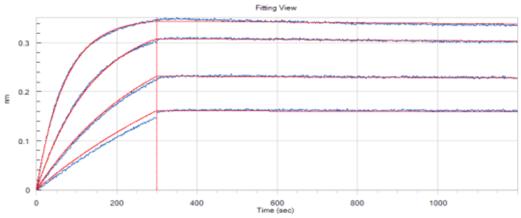

H14-AS82

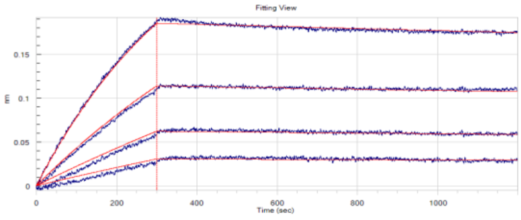

Supplementary Figure 3 3I14 scFvFc binding to recombinant HAs. Blue curves are the experimental trace obtained from biolayer interferometry experiments, and red curves are the best global fits to the data used to calculate the K_ds presented in Fig 1c.

Supplementary Figure 4

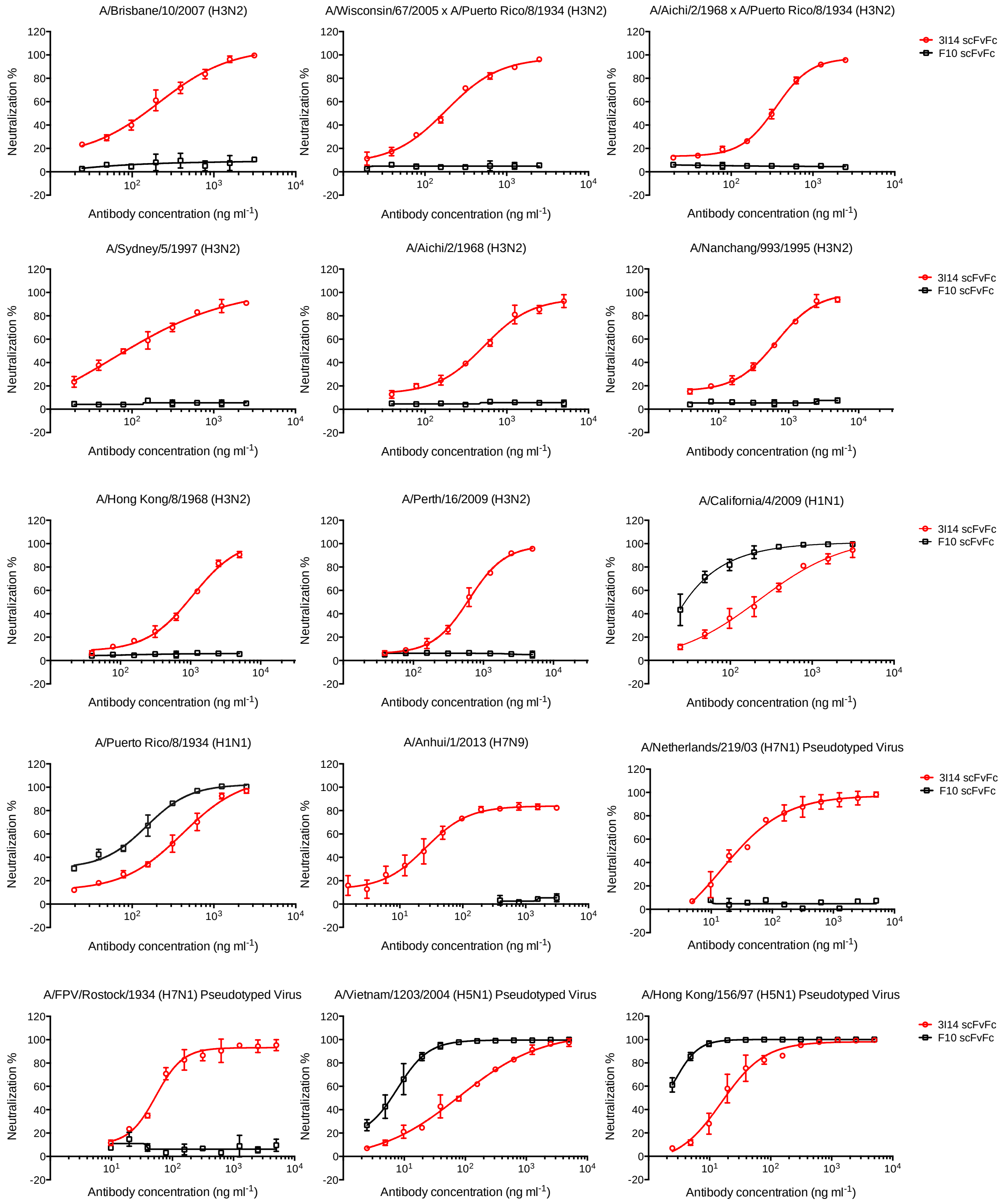

Supplementary Figure 4 3I14 scFvFc Ab neutralized influenza viruses infection and HA-pseudotyped luciferase reporter viruses. MAb 3I14 (black) and Anti-group 1 mAb F10 (red) neutralized different stains of infectious viruses and pseudotyped viruses. The data represent average neutralization titers from 2-3 independent experiments.

Figure 5

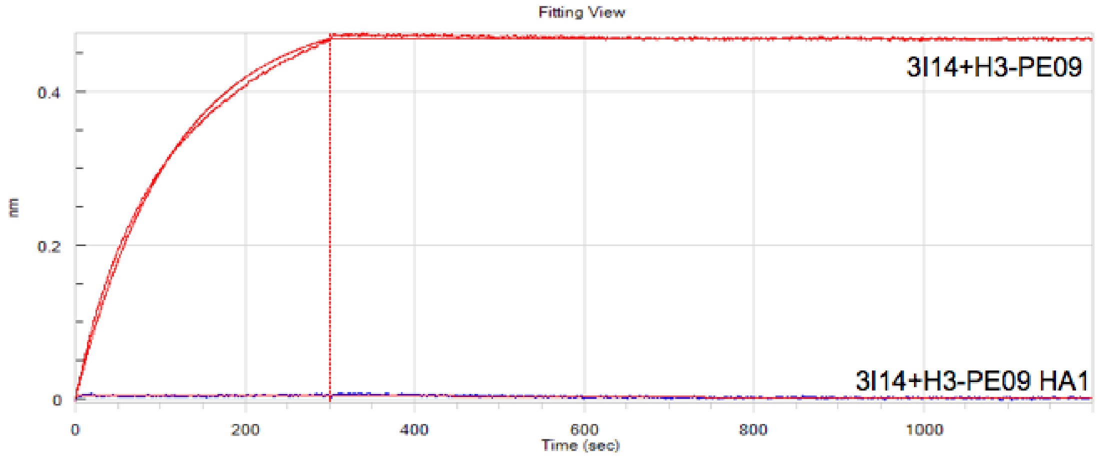

Supplementary Figure 5 3I14 scFvFc Ab binding to full-length or HA1 of recombinant H3-PE09. Blue curves are the experimental trace obtained from biolayer interferometry experiments, and red curves are the best global fits to the data. The curves show 3I14 binds H3-PE09 full-length but dose not bind H3-PE09 HA1 subunit.

Supplementary Figure 6

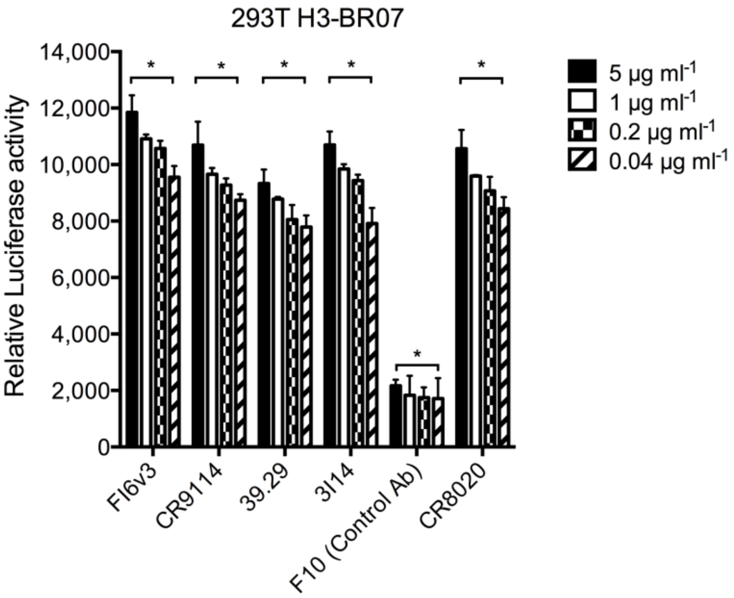

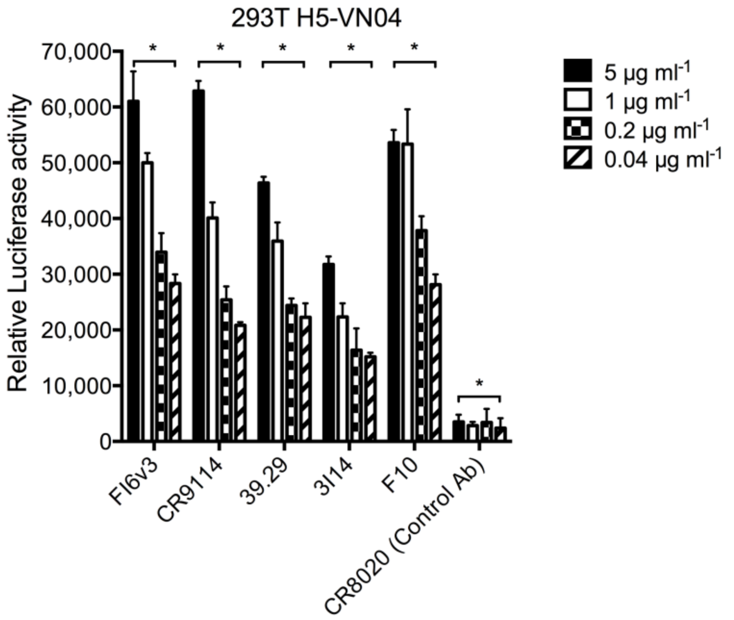

Supplementary Fig. 6 3I14 mediates a surrogate reporter-based ADCC assay

3I14 and other anti-stem bnAbs, FI6v3, CR9114, 39.29, F10 and CR8020 induced ADCC in H3- and H5- expressed 293T cells. 1×10^4/well H3 or H5-expressed 293T cells were attached to the plates prior to assay, and the medium was then replaced with low IgG serum assay buffer. Each well were added different bnAbs at concentration of 5, 1, 0.2 and 0.04 μg ml^{-1}. After one-hour, Jurkat effector cells were added for $6.0\text{x}10^4$ /well to assay plates and incubated for 6 hours. The supernatants were measured luminescence using Bio-GloTM Luciferase Assay kits (Promega). Bars represent mean ± S.E.M. *P* value was calculated using two-way ANOVA, compared to F10 (upper panel) and CR8020 (lower panel). "*" represents *p* value for each comparison < 0.0001. Data represent a representative experiment from three independent experiments, and all tests were performed in triplicate.

Supplementary Figure 7

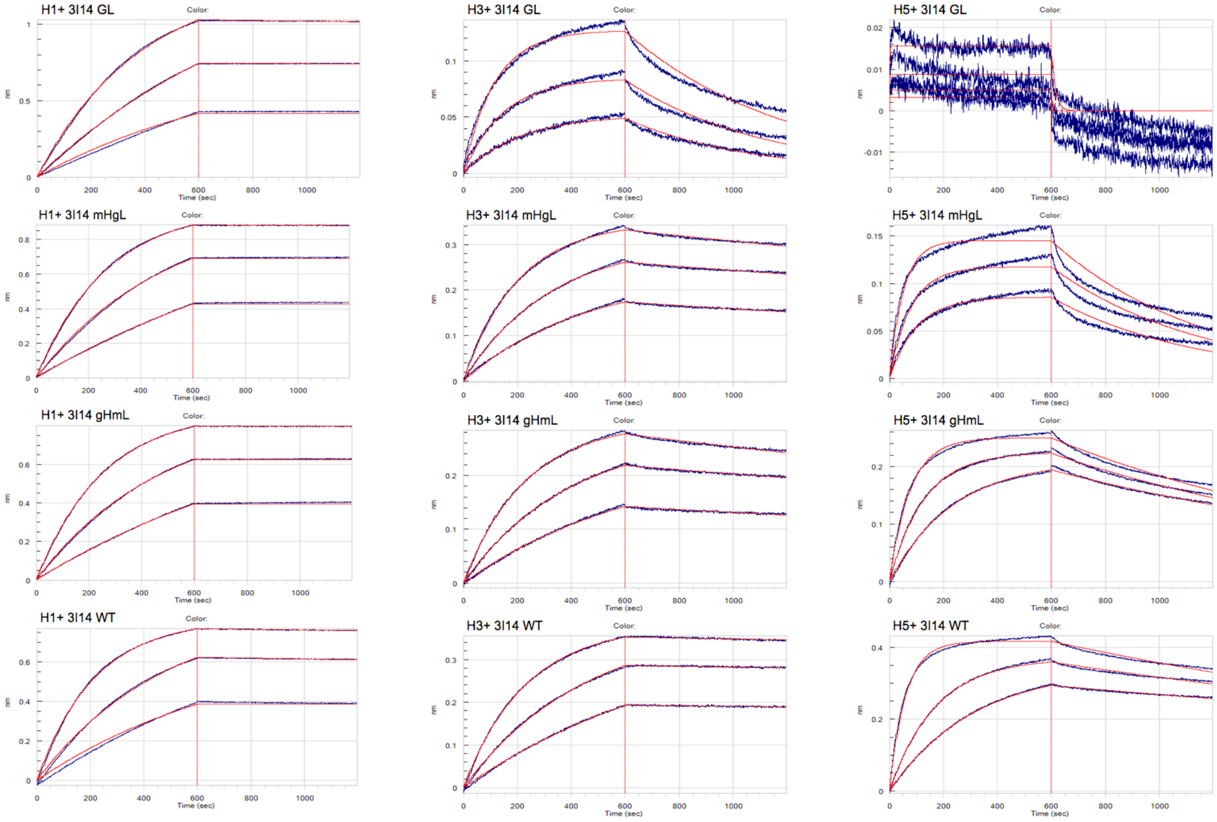

Supplementary Figure 7 Binding of the 3I14 IgG1 variants to recombinant H1, H3 and H5. Blue curves are the experimental trace obtained from biolayer interferometry experiments, and red curves are the best global fits to the data used to calculate the K_ds presented in Table 2.

Supplementary Figure 8

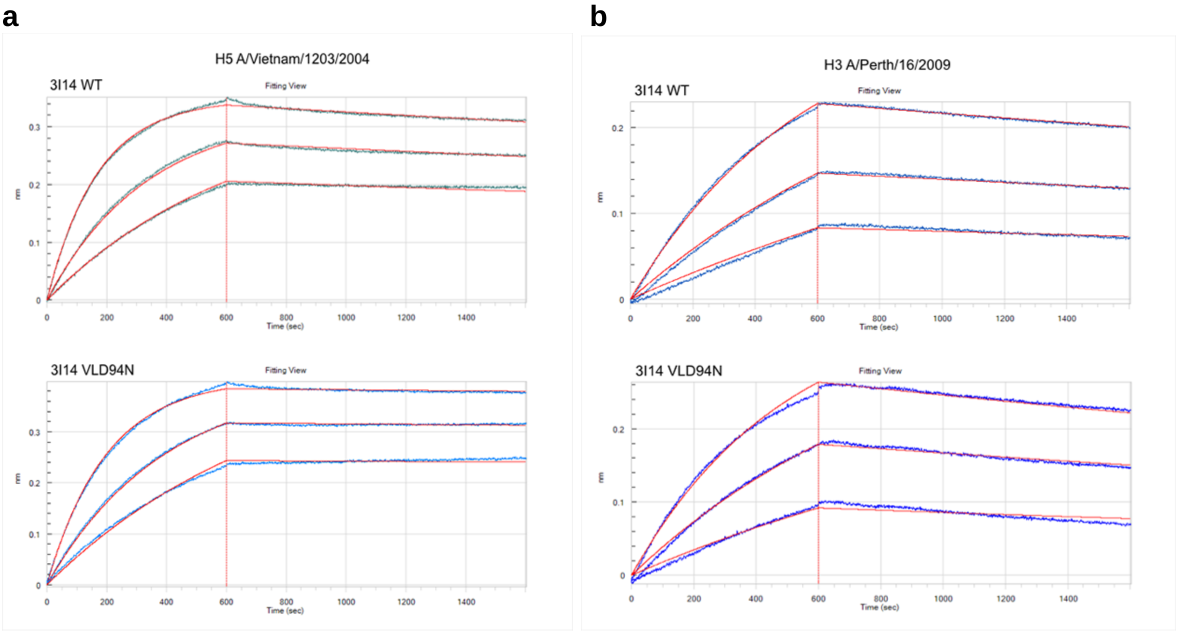

Supplementary Figure 8 3I14 WT and VLD94N IgG1 variants binding to recombinant H5-VN04 (**a**) and H3-PE09 (**b**). Green or blue curves are the experimental trace obtained from biolayer interferometry experiments, and red curves are the best global fits to the data used to calculate the K_d values. Affinity measurements (K_d values) for the binding curves were reported in Table 3.

Supplementary Figure 9

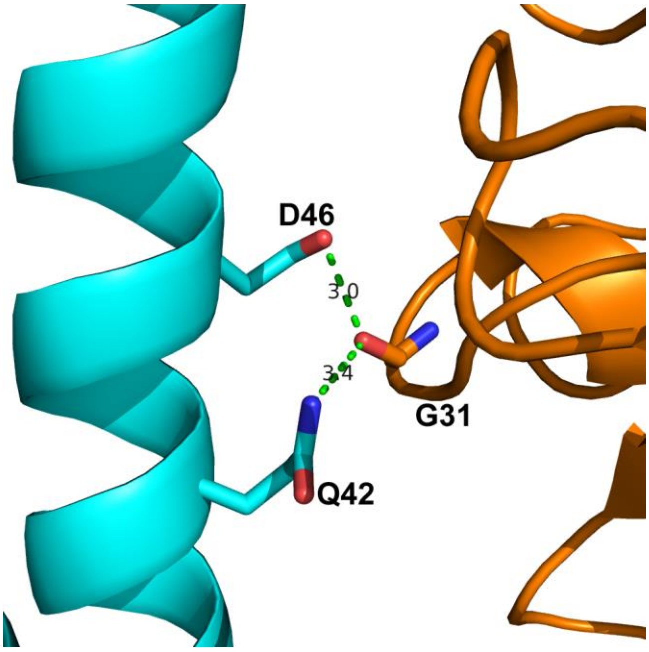

Supplementary Figure 9 The interactions of G31 of 3I14 light chain with H3 in the H3/3I14 complex model. The helix A of HA2 domain of H3 is shown as ribbon in cyan; the light chain of 3I14 is shown as ribbon in orange; the main chain atoms of G31 are shown in stick and the side chain atoms of Q42 and D46 of H3 HA2 are shown in stick; the distance between G31 and H3 are illustrated by green dash lines and labeled in black. (The PyMOL Molecular Graphics System, Version 0.99 rc6 Schrödinger, LLC).

Supplementary Figure 10

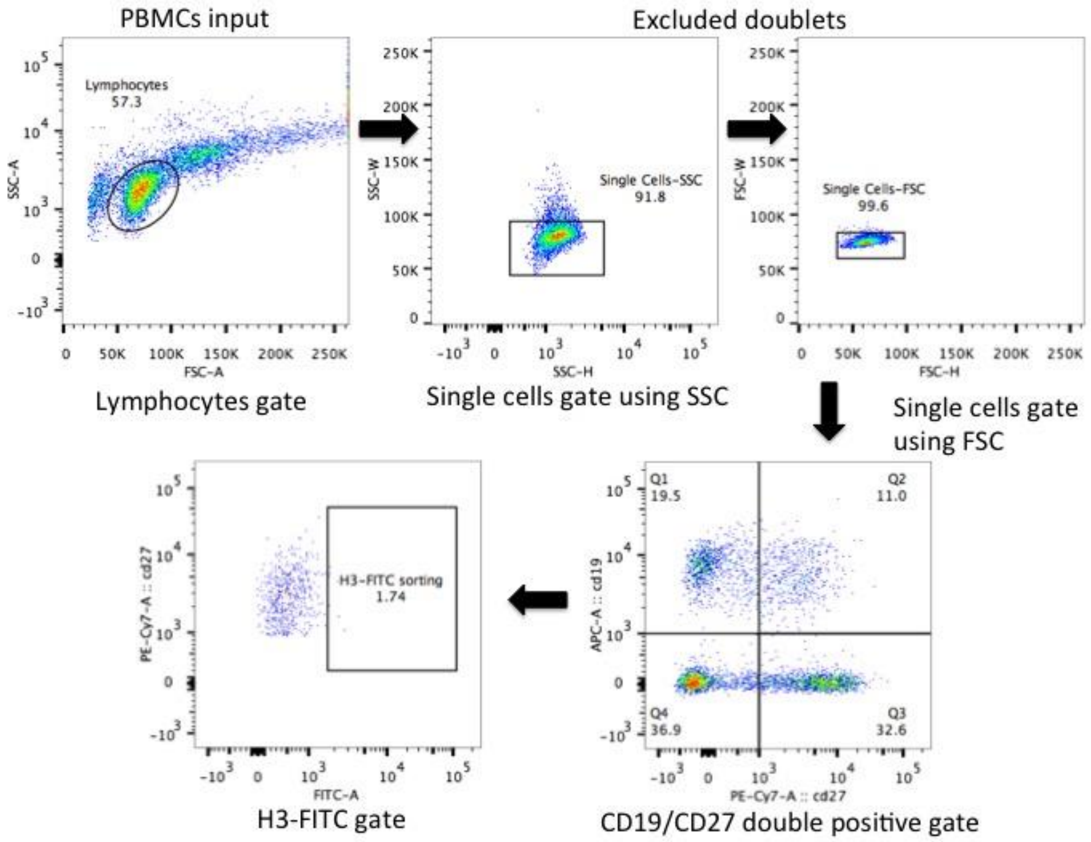

Supplementary Figure 10 The representative FACS plots for all of the gating schemes. Fresh PBMCs were sequentially gated by lymphocytes gate, SSC-H/W gate and FSC-H/W gate. The single B cells were isolated by CD19/CD27 double positive gate and H3-FITC gate and were sorted into 384-well plate.

Supplementary Figure 11

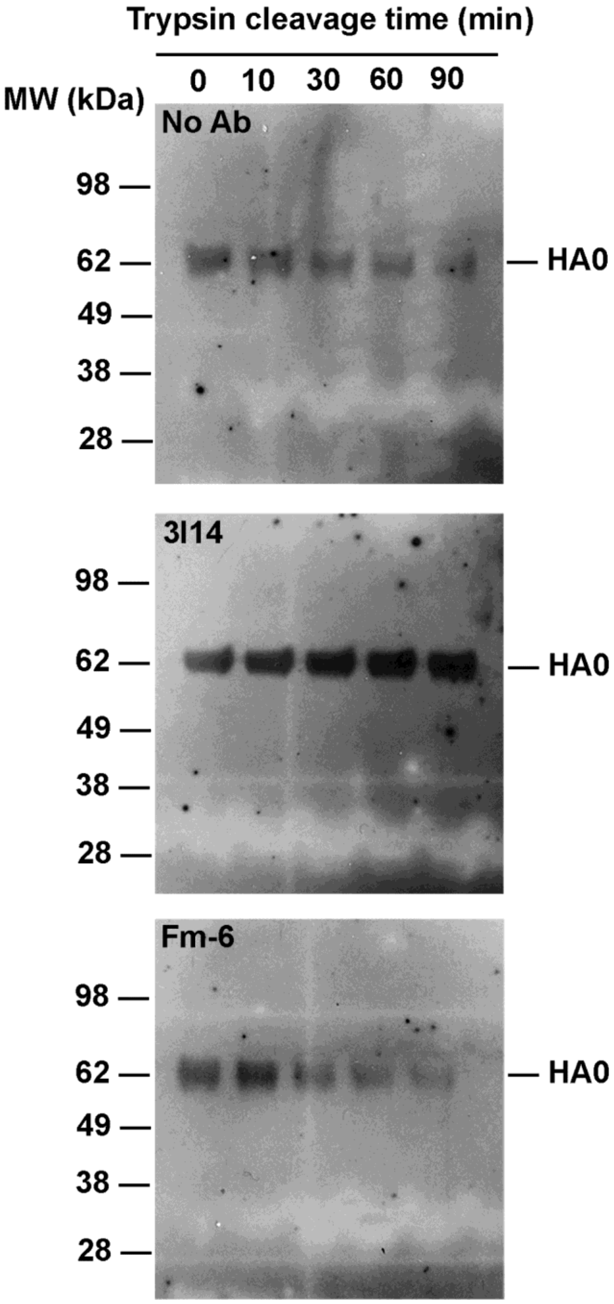

Supplementary Figure 11 Trypsin Cleavage Inhibition Assay (full-size images). 0.4 µg recombinant H3-histidine (H3-BR07) was incubated in the presence of 2.5 µg 3I14 or Fm-6 IgG1, or in the absence of antibody in Tris-HCl buffer at pH 8.0 containing 2 µg ml^{-1} Trypsin at 37°C. Trypsin digestion was stopped at several time-points by boiling the sample in a 100°C water bath. Samples were run on 10% reduced SDS-PAGE and blotted using a HisProbe-HRP Abs. Uncleaved HA (HA0) is indicated on the right side.

Supplementary Table 1 Contact residues at H3/3I14 and H5/3I14 interfaces											
**3I14**	**HCDR3**					**LCDR1**			**LCDR2**		**LCDR3**
	**Y103**	**Y104**	**F105***	**D106**	**F109***	**G31***	**N32**	**T33**	**N52**	**S53***	**D94**
H3	I18	W21<br>L38<br>I45	D19<br>L38	D19	L38	Q42<br>D46	Q42	Q42	N49		K39
H5	D19<br>I45	D19<br>K38<br>T41<br>I45	D19<br>K38	D19	K38<br>E39	Q42<br>D46	Q42		T49	N53	E39

Contact residues defined by interatomic distances < 4 Å, except residue D94 in H3 and H5 complexes defined by distances < 5 Å and < 7 Å, respectively.

The color scheme indicates contributions to the binding energy: very favorable (red); favorable (orange); neutral (blue) and unfavorable (black).

*Residues indicate the somatic mutations of germline-encoded residues.

**Supplementary Table 2 Sequence comparison of 3I14 epitope among 16 HA subtypes**																	
**Group**	**Strains**	**K_d* (nM)**	**Relative K_d** to CR9114**	**Fusion peptide**				**Helix A**									
				**18**	**19**	**20**	**21**	**36**	**38**	**39**	**41**	**42**	**45**	**46**	**49**	**53**	
Group 1	H1-CA09	0.28	-	V	D	G	W	A	L	K	T	Q	I	D	T	N	
	H1-SI06	0.03	-	V	D	G	W	A	Q	K	T	Q	I	N	T	N	
	H1-PR8	0.08	-	I	D	G	W	A	Q	K	T	Q	I	N	T	N	
	H2-JP57	-	-	V	D	G	W	A	K	E	T	Q	F	D	T	N	
	H5-VN04	1.02	-	V	D	G	W	A	K	E	T	Q	I	D	T	N	
	H5-IN05	1.05	-	V	D	G	W	A	K	E	T	Q	I	D	T	N	
	H6-NY98	-	-	V	D	G	W	A	K	E	T	Q	I	D	T	N	
	H11-MEM74	-	-	I	N	G	W	A	K	E	T	Q	I	D	T	N	
	H13-MD77	-	-	I	N	G	W	A	K	E	T	Q	I	D	T	N	
	H16-SE06	-	> 20 nM	I	N	G	W	A	K	A	T	Q	I	D	T	N	
	H8-ON68	-	-	I	D	G	W	A	Q	K	T	Q	I	D	T	N	
	H9-HK99	5.23	-	V	A	G	W	A	R	D	T	Q	I	D	T	N	
	H12-AB76	-	> 2.9 nM	V	A	G	W	A	R	D	T	Q	I	D	Q	N	
Group 2	H3-PE09	0.26	-	V	D	G	W	A	L	K	T	Q	I	D	N	N	
	H3-UY07	0.18	-	V	D	G	W	A	L	K	T	Q	I	D	N	N	
	H3-VIC11	0.33	-	V	D	G	W	A	L	K	T	Q	I	D	N	N	
	H4-NL05	0.29	-	I	D	G	W	A	L	K	T	Q	I	D	N	N	
	H14-AS82	0.29	-	I	D	G	W	A	L	K	T	Q	I	D	N	N	
	H7-NL219	0.03	-	I	D	G	W	A	Y	K	T	Q	I	D	T	N	
	H7-AH13	0.67	-	I	D	G	W	A	Y	K	T	Q	I	D	T	N	
	H10-SE02	-	-	V	D	G	W	A	Y	K	T	Q	I	D	T	N	
	H15-WA79	-	< 10 nM	I	D	G	W	A	Y	K	T	Q	I	D	T	N	

*K_d determined by Surface Plasmon Resonance (SPR) Biosensor (Fig 1c). **Relative K_d determined by Flow Cytometry (Fig 1b) and reference 16.
Residues carrying positively charged side chain are labeled orange; while negatively charged side chain residues are labeled blue.

**Supplementary Table 3 The SHMs of *IGHV1-69* and *IGHV3-30*-encoded bnAbs in V regions**			
Germline gene usage	bnAbs	SHMs in V region	
		VH	VL
IGHV3-30-encoded	3I14	11	7
	FI6	13	9
	39.29	11	11
	MAb 3.1	10	2
IGHV1-69-encoded	CR9114	17	11
	CR6261	15	7
	F10	13	5
	MAb 1.12	21	14
